# The Results of Abdominopelvic Computed Tomography Interpreted via Remote Access for the Diagnosis of Acute Appendicitis

**DOI:** 10.7759/cureus.9773

**Published:** 2020-08-16

**Authors:** Çağrı Akalın, Hadi Sasani, Nergis Ekmen

**Affiliations:** 1 General Surgery, Ordu University Training and Research Hospital, Ordu, TUR; 2 Radiology, Tekirdağ Namık Kemal University Faculty of Mecine, Tekirdag, TUR; 3 Gastroenterology, Gazi University Faculty of Medicine, Ankara, TUR

**Keywords:** acute appendicitis, computed tomography, tele-radiology

## Abstract

Introduction: Abdominal computed tomography (CT) is one of the imaging modalities for the diagnosis of acute appendicitis (AA). Today, CT scans can be interpreted via remote access called tele-radiology, besides conventional methods. The objective of this study was to evaluate the CT interpreted via tele-radiology for diagnosing AA.

Methods: In this retrospective study, a total of 679 patients, who were interpreted via tele-radiology of CT due to suspicion of AA, were evaluated. Age, gender, CT findings, pathology results and intra-operative diagnosis of those with normal CT results were analysed. A sensitivity, specificity, accuracy, positive predictive values (PPV) and negative predictive values (NPV) of CT in the diagnosis of AA were calculated.

Results: 520 patients who were operated with pre-diagnosed AA were found. Of those, 441 patients (84.8%) were diagnosed with AA according to CT reports, out of which 368 (83.4%) were positive (true-positive) and 73 (16.6%) were negative (false-positive) in terms of pathology results. In the remaining operated 79 patients with normal CT results, 58 (73.4%) were positive for AA and 21 (26.6%) (negative laparotomy) were negative for AA in terms of pathological examination. The sensitivity, specificity, accuracy, PPV and NPV of CT in the diagnosis of AA were determined as 81.2%, 67.7%, 76.7%, 83.4% and 64.2%, respectively.

Conclusion: The sensitivity and PPV rates were found similar in both conventional and tele-radiological methods. However, specificity, accuracy and NPV rates were determined lower than in literature. Additionally, the negative laparotomy rate was higher than the conventional method.

## Introduction

Acute appendicitis (AA) is a medical condition that constitutes a significant proportion of abdominal pain cases in emergency departments and requires urgent intervention [1]. Physical examination is the major component of diagnosis of AA. However, assistive radiological imaging techniques such as ultrasonography (US) and computed tomography (CT) are also utilised [2]. CT is one of the imaging methods with high sensitivity and specificity in the diagnosis of AA [3].

Tele-radiology is the transmission of images such as x-ray, CT and magnetic resonance imaging (MRI) for sharing images. Thus, on-call radiologists could interpret the images with digital devices such as smartphones or computers. Nevertheless, tele-radiology has some disadvantages such as inadequate clinical communication, medico-legal issues and quality assessment [4]. Additionally, as a result of insufficient communication between clinicians, the patient's clinical information cannot be clearly conveyed to radiologists when interpreted remotely [5]. In general, compared to conventional radiology, tele-radiology did not differ in accuracy and reliability. Today, tele-radiology is frequently used in the emergency department [6]. However, the results of CT interpreted via tele-radiology for the diagnosis of AA is not clear.

Our primary aim was to evaluate the sensitivity, specificity, accuracy, positive predictive value (PPV), negative predictive value (NPV) and negative laparotomy (NL) rate interpreted via tele-radiology of CT scans of the abdomen for diagnosing AA.

## Materials and methods

Study design

A study was designed retrospectively and ethical approval for the study was obtained from the clinical research ethics committee of the institution (Approval Number: 2019/80). Between January 2014 and March 2019, patients who underwent CT interpreted via remote access due to suspicion of AA analyzed in the emergency department at high-volume two-center hospitals were analyzed. Age, gender, CT findings, pathology results and intraoperative diagnosis of those with normal CT results were recorded. Patients with suspected AA were evaluated by practitioners and emergency doctors (specialists). The CT scans and operations were performed in the same hospital. Patient information was obtained from the hospital information system and patient files.

Patient selection

The inclusion criteria were: 1) over 18 years of age, 2) remotely evaluated CT examination and 3) diagnosis of AA confirmed by CT scan defined by the following criteria: (a) an enlarged appendix exceeding 6 mm in diameter cecal wall thickening (more than 2 mm), (b) enhancing wall and peri-appendicular edema and/or minor fluid collection, (c) intraluminal appendicolith (>3 mm stone within appendix) and (d) peri-appendicular gas or fluid, abscess, phlegmon and plastron.

The exclusion criteria were as followings: 1) under 18 years of age, 2) non-remotely evaluated CT examination, 3) without administration of intravenous (IV) contrast materials during scanning, 4) CT images with poor quality or artifacts and 5) operated with a preliminary diagnosis of appendicitis with only US results.

Scanning protocol and interpreted via remote access

The field of view of the abdominopelvic region in this study was located between the xiphoid process of the sternum and the pubic symphysis. CT examinations were performed on 16-slice CT scanners (Alexion 16, Toshiba Medical Systems, Japan). The following CT protocol was used: 120 kVp, a tube current of 150-165 mAs, maximum 2.5 mm collimation, a slice thickness of 3 mm and a 0.5 s rotation time. A dynamic image with arterial (scanning delay, 20-30 s), portal venous (scanning delay, 60-70 s) and equilibrium (scanning delay, 2-3 min) phases were obtained after injection of a total of 50-100 ml contrast material (Omnipaque® 300-350 mg/50-100 milliliters, Opakim) containing a concentration of 300-350 mg/mL at 4 mL/s velocity. No rectal or oral contrast material was administered. During CT scanning, images in axial, sagittal and coronal planes were obtained with contiguous 5-mm slices. Interpretation of the abdominal CT studies was performed by radiologists, contracted by the hospitals, using picture archiving and communication system (PACS). The radiologists made a diagnosis after reviewing scans. The reviewer evaluated the images on another independent workstation.

Pathologic analysis

The pathology specimens were interpreted by pathologists at the same hospital. In the pathological evaluation, any findings, such as edema, inflammation, necrosis and abscess, were considered pathologically "positive"; the lack of these findings was considered "negative". Pathology results for the diagnosis of AA were categorized as "positive" or "negative".

Rates

**Figure 1:**
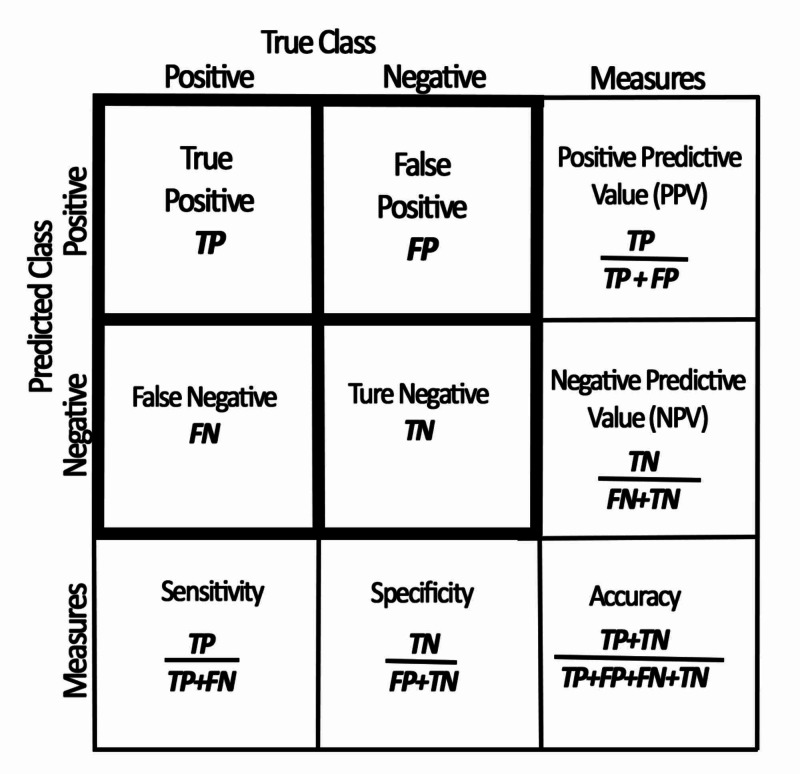
Calculation of sensitivity, specificity, accuracy, positive and negative predictive values TP: True Positive; TN: True Negative; FP: False Positive; FN: False Negative

Sensitivity, specificity, accuracy, PPV and NPV of the patients were performed according to the calculations shown in Figure 1 [7]. Negative laparotomy (NL) rate was considered as the ratio of patients with normal histopathology or macroscopy to patients undergoing laparotomy [8].

Statistical Analysis

Data were analyzed using statistical software SPSS version 20 (IBM, Chicago, USA). Descriptive statistics for continuous variables - mean, standard deviation, minimum and maximum values - were expressed as number and percentage for categorical variables. The data distribution was evaluated using the Kolmogorov-Smirnov test. Chi-square test was used in determining the relationship between categoric variables. Mann-Whitney U and Kruskal-Wallis tests were performed in relation to continuous variables. P-value <0.05 was considered as statistically significant.

## Results

Out of a total of 679 patients who underwent CT due to the suspicion of AA, 520 patients that were operated with pre-diagnosed AA were found. Their mean age was 38.87±17.49 years (range 18-93). Overall, 356 (52.4%) of the patients were female and 323 (47.6%) were male. Additionally, 238 (35.1%) of the patients had negative CT findings for AA, and 441 (64.9%) had positive. A total of 520 patients were operated upon, including 441 patients with CT results compatible with AA and 79 patients with normal CT results but suspected upon physical examination. The pathology results of the 441 patients with positive CT scans were evaluated for AA - 368 (83.4%) (true-positive) were AA positive and 73 (16.6%) (false-positive) were negative for AA. The results of patients with false-positive are given in Table 1.

**Table 1:** The results of patients with false-positive

Diagnosis	n=73
Gynaecological	
Ovarian cyst	8
Pelvic inflammatory disease	7
Endometriosis	1
Urological	6
Gastrointestinal	
Inflammatory bowel disease	4
Meckel's diverticulitis	2
Enteritis	5
Malignancy	2
Other	
Mesenteric lymphadenitis	7
Omental torsion	2
Panniculitis	1
Normal	28

Of the operated 79 patients with normal CT result for suspected AA, 58 (73.4%) were detected with AA, but in 21 (26.6%) (negative laparotomy) of the cases, AA was not detected. The break-up of the patients with negative laparatomy (NL) (n=21) were as following: ovarian cyst rupture (n=8), diverticulitis (n=5), panniculitis (n=1), inflammatory bowel disease (n=2), non-specific abdominal pain (n=3) and gastrointestinal cancer (n=2). Table 2 shows the sensitivity, specificity, accuracy, PPV and NPV of the CT scans in the diagnosis of AA.

**Table 2:** The sensitivity, specificity, accuracy, positive and negative predictive values of CT in diagnosis of acute appendicitis *Additionally, the pathology result of unoperated healthy patients was considered normal CT: computed tomography

		Patients with suspected acute appendicitis (n=679)			
		Condition positive (As confirmed on pathology) (n=453)	Condition negative (As confirmed on pathology)* (n=226)		
CT (n=679)	Positive (n=441)	True-positive (n=368)	False-positive (n=73)	Positive predictive value (368/441) 83.4%	
					Negative (n=238)	False-negative (n=85)	True-negative (n=153)	Negative predictive value (153/238) 64.2%	
			Sensitivity (368/453) 81.2%	Specificity (153/226) 67.7%					Accuracy (368+153/368+73+85+153) 76.7%	

## Discussion

AA occurs in around 7% population worldwide [9]. The diagnosis of AA is crucial as it may lead to a poor prognosis if not treated surgically, which is associated with increased morbidity and mortality [10]. In the diagnosis of AA, physical examination alone holds 76-80% of the diagnostic rate, while the NL rate is around 20%. The inclusion of assistive imaging methods such as US and CT in addition to the physical examination has increased the rate of diagnosis and has lead to a decline in the rate of NL [10,11]. Additionally, in a large-scale retrospective analysis by Shaligram et. al., they examined interpreted CT studies in a total of 13,288 young male patients (18-55 years of age) with suspected AA via tele-radiology [12]. The authors concluded that the use of the CT examination is associated with improved postoperative complications and lower cost. On the other hand, Hains et al. reported that patient information was contradictory to the CT examination interpreted via tele-radiology [13], and insufficient transmission in regard to patient information caused stress among the radiologists. Thus, this situation results in the increment of the workload among radiologists and posed a risk for accurate diagnosis and patient safety [5]. We think that these factors may affect CT interpretation negatively.

It is well known that due to being radiation-free, cost-effective and mostly available in the emergency services, US is the first-line imaging modality to provide a demonstration of AA and possible complications [14]. However, depending on the operator and patient-related factors, US can be inconclusive and it may require a further investigation such as cross-sectional modality like abdominal CT. According to clinical history and physical examination findings, in the suspected AA cases, abdominal CT can be performed with or without contrast material administration (IV, oral/rectal). IV contrast administration is useful in the demonstration of wall enhancement and the assessment of complications (abscess, perforation). Although oral/rectal contrast administration can be used to show the obstruction of the appendix, using enteric contrast material is unnecessary owing to no increase in sensitivity or specificity in AA [15] Also, CT is used in showing of the AA located retrocecally. In such cases, it may result in the formation of abscess in the pararenal space as well as spreading to the liver nude-area [16]. 

In cases of suspicion in the diagnosis of AA, surgeons decide on laparotomy in order to protect the patient from possible complications and to protect themselves from possible medico-legal processes. However, NL is always regarded as a probable situation and leads to prolonged duration of hospital stay and increased morbidity [17,18]. The use of CT for suspected AA was made obligatory in the Netherlands in 2008, which lead to a decrease in the rate of NL from 19% to 5% in three years [14,19]. In Netherlands, US examination is performed as the first step for diagnosing AA, and CT is performed only in patients with suspected AA after US examination. Additionally, they reported that a delay of imaging and the radiation exposure of CT being balanced against the risks of unnecessary surgery has been the subject of controversy. The use of many clinically unnecessary CT scans has been reported to result in more radiation exposure [20]. In order to reduce this exposure, the combination of US and low-dose CT scan is of the most value [21]. In a meta-analysis conducted by Dahlberg et al. in 2014, it was stated that the NL rate declined from 10% to 1.7% within 10 years with the use of assistive radiological imaging methods in the diagnosis of AA [22]. In this study also, US examination was performed as the first step for diagnosing AA. Unfortunately, the common features of these studies are the lack of standard conditions on premedication, communication between clinicians and using a contrast drug for CT examination. In our study, the NL rate was found to be 26.6%, which is higher compared to the literature. We think that performing CT in suspected AA cases after US examination protects patients from unnecessary radiation exposure.

CT scans were interpreted via conventional methods by a radiologist before using tele-radiology. In the literature, there are many studies about the information of conventional methods for diagnosing AA. In 1986, Balthazar et al. reported that CT could be useful in the diagnosis of AA [23]. In 1991, they carried out another study in which the CT scan with IV contrast revealed a 98% sensitivity, 83% specificity and 93% accuracy in the diagnosis of AA [24]. The CT scan for the diagnosis of AA can be performed without using oral or rectal contrast, but with or without IV contrast material. Malone et al. reported the sensitivity of the CT scan without contrast in the diagnosis of AA as 87%, specificity as 97%, accuracy as 93%, PPV as 94% and NPV as 93% [25]. In 2015, Atema et al. reported the sensitivity of CT with IV contrast as 95%, specificity as 87%, the accuracy, PPV and NPV as 92% in patients with AA [26]. In our study, we only evaluated the results of patients undergoing CT with IV contrast. CT scans in the mentioned studies were not interpreted via tele-radiology and the radiologists had clinical knowledge about AA. The sensitivity and PPV rates of both contrast-enhanced and non-enhanced CT for the diagnosis of AA of these studies were found to be similar in our study. However, the specificity, accuracy and NPV were determined generally lower than in the literature.

Choudhri et al. evaluated the ability to diagnose AA from CT using tele-radiology, and 25 CT scans were interpreted by five radiologists using an iPhone [27]. The authors found that 88% appendicolith were detected, and the false-positive rate was 95%. They found no differences between PACS and iPhone readings. Another study by Choi et al. investigated interpretation via ultramobile computer and liquid crystal display (LCD) of CT scans for the diagnosis of AA [28]. Additionally, in the study by Choi et al., a total of 100 abdominal CT scans was presented to four radiologists and the sensitivity and specificity rates were found as 87-95% and 84-100%, respectively. In our study, the specificity rate was similar, but the sensitivity rate was slightly lower. Kim et al. evaluated the feasibility of smartphone-based remote-control system with LCD monitor of a PACS in 120 cases that showed 60 of the patients had signs of appendicitis [29]. An evaluation of AA was done by 16 board-certified emergency physicians as raters. The average areas under the receiver operating characteristic curve for all readers with smartphone and LCD monitor were 0.978 and 0.974 respectively. There was no significant difference in the diagnostic performance between the two devices (p=0.55), and the inter-rater agreement for each device was rated as very good (the kappa value for the smartphone was 0.809 and for the LCD monitor was 0.817) [29]. In the present study, routinely used diagnostic monitors were used. Additionally, we could compare the results only with the literature due to the lack of a control group.

There are some limitations of the current study. Firstly, this study was designed retrospectively and conducted with a limited number of patients. Secondly, there was no control group in this study for comparison. Thirdly, our study involved a single consultation system, two hospitals, a standard-dose CT protocol, and only a few off-site radiologists, which may limit the generalizability of the study. Another limitation of this study is that some parameters, such as the workload and experience of the radiologists and inadequate communication between the clinicians may have affected the results of the CT reports. These parameters could not be included in the study due to having limited information in that regard. We suggest that future studies focus not only on digital features but also on these parameters. Despite these limitations, the limited number of studies reported in the literature is an advantage for the current study. Moreover, healthy patients who underwent the CT examination due to the suspicion of AA were also included. Thus, the specificity, accuracy and NPV rates could be calculated in the present study.

## Conclusions

The sensitivity and PPV rates of CT scans interpreted for diagnosing AA were found to be similar in both conventional and tele-radiological methods. However, the specificity, accuracy and NPV rates were determined lower than in literature. Additionally, the NL rate was higher than the conventional method.
